# Pragmatic Phenotype–Electrophysiology–Genomics Integration in Pediatric Congenital Myasthenic Syndromes: Insights From 36 Patients in a Single‐Center Study in China

**DOI:** 10.1002/cns.71123

**Published:** 2026-09-05

**Authors:** Liya Cui, Kaiyue Ma, Xiaona Fu, Lin Ge, Bingbing Jia, Wenna Ma, Lei Yang, Zhimei Liu, Junlan Lv, Wenmiao Xu, Lijuan Wang, Guangyuan Zhao, Jiuwei Li, Jie Wu, Quan Wang, Changhong Ding, Xiaotun Ren, Xinming Shen, Hui Xiong

**Affiliations:** ^1^ Department of Neurology Beijing Children's Hospital, Capital Medical University, National Center for Children's Health Beijing China; ^2^ Bio‐X Institutes, Key Laboratory for the Genetics of Developmental and Neuropsychiatric Disorders Ministry of Education, International Peace Maternity and Child Health Hospital, Shanghai Key Laboratory of Embryo Original Diseases, School of Medicine, Shanghai Jiao Tong University Shanghai China; ^3^ Pediatric Intensive Care Unit Beijing Children's Hospital, National Center for Children's Health, Capital Medical University Beijing China; ^4^ Department of Emergency Beijing Children's Hospital, Capital Medical University, National Center for Children's Health Beijing China; ^5^ Department of Neurology Mayo Clinic, Mayo Clinic College of Medicine Rochester Minnesota USA

**Keywords:** congenital myasthenic syndrome, genotype‐guided therapy, neuromuscular transmission, pediatric neuromuscular disorders, repetitive nerve stimulation, variants of uncertain significance

## Abstract

**Aims:**

To characterize the clinical, electrophysiological, and genetic spectrum of pediatric CMS and evaluate genotype‐informed outcomes using an integrated phenotype–electrophysiology–genomics approach.

**Methods:**

We retrospectively reviewed 36 pediatric CMS patients evaluated at a single center between 2015 and 2025. Clinical features, RNS, targeted NGS/WES variants, ventilator use, treatments, ACMG/AMP classifications, and MG‐ADL outcomes were analyzed.

**Results:**

Of 36 patients, 28 (77.8%) developed symptoms in the neonatal period or infancy. Biallelic variants involved 17 CMS genes; postsynaptic CMS was most common (55.6%, 20/36). *COLQ* and *CHRNE* were the most frequent genes (13.9%, 5/36 each), followed by *CHAT* (11.1%, 4/36). VUS were detected in 19 patients (52.8%, 19/36), including 8 with biallelic VUS supported by phenotype, neuromuscular transmission findings, treatment response, and follow‐up. RNS showed a ≥ 10% decrement in 16/21 tested patients (76.2%). *CHAT*‐CMS was associated with higher ventilator use (3/4 vs. 6/32; *p* = 0.041) and early mortality (3/4 vs. 1/32; *p* = 0.002). Median MG‐ADL improved from 5 to 3 after genotype‐informed therapy.

**Conclusion:**

Pediatric CMS shows marked genetic heterogeneity and frequent VUS‐related uncertainty. Integrating phenotype, electrophysiology, and genomics supports diagnosis and mechanism‐guided therapy. *CHAT*‐CMS is high risk for early respiratory failure and mortality.

## Graphical abstract

In 36 Chinese pediatric CMS patients, integrated phenotype, RNS, and genomic assessment revealed marked genetic heterogeneity across 17 CMS‐associated genes and frequent VUS‐related uncertainty. Genotype‐informed therapy improved MG‐ADL scores, while *CHAT*‐CMS identified a high‐risk subgroup for early respiratory failure and mortality.

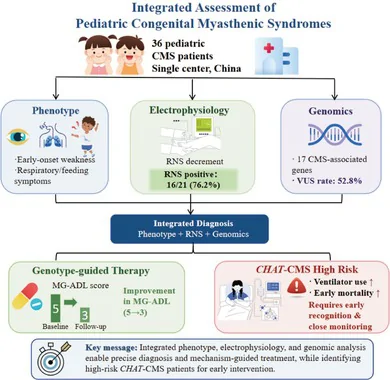

## 1 Introduction

Early‐onset neuromuscular disorders pose substantial diagnostic challenges in pediatric practice. Phenotypic overlap between congenital and acquired neuromuscular diseases is common, particularly in neonates and infants, in whom hallmark features such as fatigable or fluctuating weakness may be absent, subtle, or difficult to recognize [1]. Electrophysiological assessments, including repetitive nerve stimulation (RNS), are often challenging in young children. This is due to limited cooperation, developmental immaturity of neuromuscular transmission, and technical constraints related to body size and electrode placement. Meanwhile, the broader adoption of next‐generation sequencing (NGS) has improved diagnostic yield but has also increased the detection of variants of uncertain significance (VUS), creating new challenges for variant interpretation and clinical decision‐making. Currently, pediatric neuromuscular diagnosis often depends on incomplete or indirect evidence, underscoring the need for pragmatic, integrative approaches that combine clinical, electrophysiological, and genomic data in real‐world care.

Congenital myasthenic syndromes (CMS) are a heterogeneous group of inherited disorders caused by impaired neuromuscular junction (NMJ) function and defective neuromuscular transmission. CMS typically presents in infancy or early childhood with fluctuating fatigable weakness affecting ocular, bulbar, axial, respiratory, and limb muscles. Pathogenic variants in more than 40 genes have been implicated, affecting presynaptic neurotransmitter synthesis/release, synaptic basal lamina and acetylcholinesterase anchoring, postsynaptic acetylcholine receptor (AChR) function, and pathways involved in glycosylation and NMJ development [2, 3, 4]. Reported prevalence varies across regions, ranging from approximately 1.8 to 22.2 per 1,000,000 individuals [3, 5, 6, 7, 8]. However, epidemiologic and clinicogenetic data from China, especially in pediatric CMS, remain limited.

Because its clinical features overlap with numerous congenital and acquired neuromuscular disorders, CMS is frequently underrecognized, resulting in delayed diagnosis and suboptimal management. In children, early signs such as hypotonia, delayed motor development, limb‐girdle weakness, and recurrent respiratory or feeding problems may mimic muscular dystrophies, congenital myopathies, or spinal muscular atrophy (SMA), while fluctuating weakness with ocular or bulbar involvement can resemble acquired myasthenia gravis (MG) [2]. Importantly, the distinct molecular mechanisms underlying CMS are linked to divergent, and in some cases opposing, treatment strategies. For example, cholinesterase inhibitors (ChEIs) may be effective in certain postsynaptic CMS subtypes, such as *RAPSN*‐ and *CHRNE*‐related CMS, but may be harmful and should generally be avoided in *COLQ*‐related CMS and slow‐channel CMS. Patients with *AGRN*‐, *LRP4*‐, *MUSK*‐, or *DOK7*‐related CMS usually show limited or no response to ChEIs, although these agents are not strictly contraindicated. β_2_‐Adrenergic agonists may be effective across multiple CMS subtypes and are particularly emphasized in *COLQ*‐ and *DOK7*‐related CMS, because ChEIs may be detrimental in *COLQ*‐related CMS and are usually ineffective in *DOK7*‐related CMS. Consequently, misinterpretation at the genetic or mechanistic level may result not only in diagnostic delay but also in inappropriate therapy, potentially causing direct clinical harm [1, 4, 9]. However, because most clinical centers lack the facilities or on‐site support to perform functional expression studies for variants in CMS‐related genes, establishing a standardized integrated clinicogenetic analysis protocol is essential. Such an approach enables accurate interpretation of genetic findings in the context of clinical phenotypes, thereby facilitating precise diagnosis and guiding mechanism‐based treatment strategies in CMS.

Here, we report a clinicogenetic analysis of 36 pediatric CMS patients at a tertiary referral center in China. Given the limited feasibility of electrophysiology in early childhood and the high prevalence of VUS in genomic testing, we integrated phenotyping results, RNS, and molecular findings to support pragmatic diagnostic assessment and mechanism‐informed treatment in pediatric practice. We highlight clinical scenarios in which CMS should be prioritized—particularly in neonates and young infants with feeding or respiratory difficulties, hypotonia, delayed motor development, and infection‐associated worsening of weakness—and illustrate how phenotype–neurophysiology integration can mitigate VUS‐related uncertainty to enable provisional diagnosis and timely management.

## 2 Materials and Methods

### 2.1 Diagnostic Criteria

In the absence of formal consensus diagnostic criteria for CMS, we applied an experience‐based operational framework integrating clinical, electrophysiological, and genetic evidence to standardize diagnostic confidence and support clinical decision‐making in pediatric practice.

Inclusion Criteria: (A) Clinical features: Manifestations of fluctuating muscle weakness, neonatal apnea, dysphagia, or reduced limb activity. MRC grading (0–5) was recorded when feasible; otherwise, weakness was documented by affected muscle group. (B) Electrophysiological findings: evidence of impaired neuromuscular transmission on electrophysiological testing, including a decrement of ≥ 10% in compound muscle action potential (CMAP) amplitude on low‐frequency repetitive nerve stimulation (RNS), abnormal jitter or blocking on single‐fiber electromyography when available, or other electrophysiological findings consistent with a neuromuscular junction transmission defect. (C) Genetic findings: identification of variant(s) in CMS‐associated genes consistent with the expected mode of inheritance. Genetic findings were further categorized as: (C1) pathogenic or likely pathogenic (P/LP) variant(s); and (C2) variant(s) of uncertain significance (VUS).

Exclusion Criteria: (a) Acute Etiologies: Acute muscle weakness attributable to conditions such as rhabdomyolysis, acute intoxication, or botulism. (b) Other genetic neuromuscular disorders: Confirmed diagnosis of congenital myopathies, congenital muscular dystrophy, or SMA. (c) Acquired Neuromuscular Disorders: including MG, inflammatory myopathies, or endocrine myopathies. (d) Significant Comorbidities: Presence of other severe concurrent illnesses, such as malignancy or significant impairment of cardiac, hepatic, or renal function.

**FIGURE 1:**
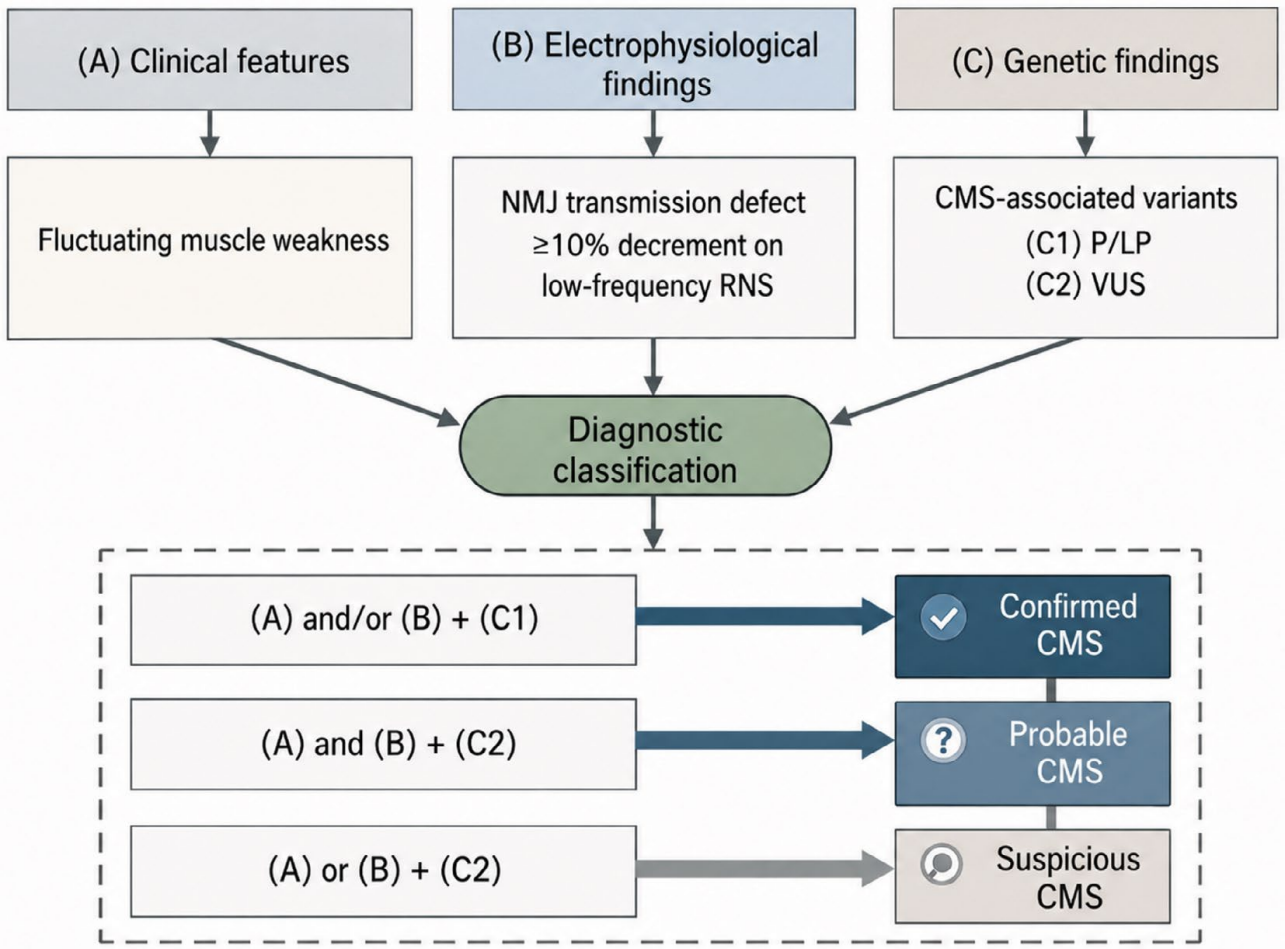
Diagnostic workflow for CMS. Diagnostic classification was based on clinical features (A), electrophysiological findings (B), and ACMG‐based interpretation of CMS‐associated variants (C). Confirmed CMS was defined as (A) and/or (B) plus P/LP variant(s) (C1); Probable CMS as (A) and (B) plus VUS (C2); and Suspicious CMS as (A) or (B) plus VUS (C2). ACMG, American College of Medical Genetics and Genomics; CMS, congenital myasthenic syndromes; NMJ, neuromuscular junction; P/LP, pathogenic/likely pathogenic; RNS, repetitive nerve stimulation; VUS, variant of uncertain significance.

Diagnostic Classification: A diagnosis of confirmed CMS was assigned when a patient met the clinical and/or electrophysiological criteria [(A) and/or (B)] and harbored P/LP variant(s) consistent with the expected mode of inheritance (C1). A diagnosis of probable CMS was assigned when both the clinical and electrophysiological criteria [(A) and (B)] were met and only VUS were identified (C2). A diagnosis of suspicious CMS was assigned when either the clinical or electrophysiological criterion [(A) or (B)] was met in the presence of VUS (C2). The diagnostic workflow is illustrated in Figure 1.

### 2.2 Genetic Analyses

Genetic testing was performed in certified clinical laboratories (Chigene [Beijing], Fuzhou Frey Medical Laboratory, MyGenostics, and Beijing Kangso Medical Inspection), all of which are accredited by the College of American Pathologists (CAP) and follow rigorous clinical‐grade quality‐control procedures. Genomic DNA was extracted from peripheral blood. Of the 36 patients, 5 underwent targeted NGS panels (Table S1) for neuromuscular/CMS‐associated genes, while the remaining cases were assessed with whole‐exome sequencing (WES). Copy‐number variant (CNV) calling from NGS/WES data was performed when applicable, and CNVs were confirmed by quantitative PCR (qPCR) in all relevant cases. Variants prioritized for clinical reporting were confirmed, and parental samples were obtained for segregation analysis in all families—either through trio‐based testing in a subset or by targeted parental testing after proband sequencing—typically using Sanger sequencing (or orthogonal methods as appropriate for CNVs).

Sequence data were analyzed against the GRCh37/hg19 reference genome. Variant calls were subsequently liftovered to the GRCh38 reference genome for reporting in Table S2. Variants were annotated using population frequency resources (dbSNP/1000 Genomes, gnomAD including East Asian subsets, ExAC/ExAC‐EAS, and ESP6500) and clinical databases (ClinVar and OMIM), supplemented by literature review and in silico prediction metrics reported by the testing laboratories (e.g., REVEL, CADD, AlphaMissense, SpliceAI, and conservation/splice predictors). Variant interpretation followed ACMG/AMP criteria, incorporating population frequency, predicted functional consequence including loss‐of‐function where applicable, segregation and allelic data, and phenotype/electrophysiology consistency. Variants are reported in Table S2 using HGVS transcript‐ and protein‐level nomenclature when applicable. For exon‐level deletions and other CNVs, descriptive notation is used when standard cDNA‐level HGVS expressions are not applicable.

### 2.3 Participants

Between January 2015 and June 2025, 55 patients who presented to the inpatient wards and/or outpatient clinics of Beijing Children's Hospital, affiliated with Capital Medical University, were clinically suspected of having CMS before genetic testing. Following evaluation according to the predefined inclusion and exclusion criteria, 36 patients with CMS‐associated variants consistent with the expected mode of inheritance were included in the final cohort, whereas 19 patients were excluded because they did not meet the eligibility criteria.

Baseline data were collected for these patients, encompassing demographic information, clinical manifestations, birth history, personal history, family history, physical examination findings, auxiliary examinations, genetic results, and treatment details.

### 2.4 Electrophysiology

RNS was performed in patients who were able to tolerate and complete the examination. The studies were conducted on multiple nerves, including the facial, spinal accessory, axillary, ulnar, and common peroneal nerves. Under standard stimulation rates of 1, 3, and 5 Hz, a neuromuscular transmission defect was defined as a decrement of ≥ 10% in the compound muscle action potential (CMAP) amplitude between the first and the fifth stimuli [10]. In a subset of patients, high‐frequency stimulation of the ulnar nerve was additionally performed at 10 and 20 Hz to assess for presynaptic facilitation. An incremental response was defined as an increase of > 100% in CMAP amplitude between the first and final stimuli. No incremental responses were observed in this cohort.

### 2.5 Follow‐Up

All patients were followed longitudinally in the outpatient clinic, with the final follow‐up completed in November 2025. This study employed the Myasthenia Gravis Activities of Daily Living Scale (MG‐ADL) score to comprehensively assess patients' functional status at both diagnosis and their final follow‐up visit. The evaluation covered multiple specific aspects, including speech, chewing and swallowing function, the ability to brush teeth independently, squatting and standing movements, ptosis, and the presence of diplopia. MG‐ADL scores range from 0 to 24, where higher scores indicate greater disease severity. The specific values of MG‐ADL scores at diagnosis and the final follow‐up visit are provided in Table 1.

**TABLE 1:** Clinical characteristics of 36 children with congenital myasthenic syndrome.

Subject	Sex	Gene	Onset age (months)	Age at diagnosis (months)	Ventilator usage	RNS	Family history	Treatment	Survival status	Follow‐up duration (months)	MG‐ADL^a^	MG‐ADL^b^
1	M	*CHAT*	4	7	Y	NP	N	Py	Deceased	NA	NA	NA
2	M	*CHAT*	1	15	Y	−	N	Py	Deceased	NA	NA	NA
3	M	*CHAT*	0	4	Y	NP	N	N	Deceased	NA	NA	NA
4	M	*CHAT*	36	36	N	NP	N	Py	Alive	44	4	3
5	M	*SLC5A7*	0	23	N	+	N	Py	Alive	84	7	2
6	F	*COLQ*	0	13	N	NP	Y	Sal	Alive	52	5	2
7	F	*COLQ*	0	13	N	NP	N	N	LTFU	NA	NA	NA
8	M	*COLQ*	0	7	N	NP	N	N	LTFU	NA	NA	NA
9	M	*COLQ*	0	94	N	+	N	Sal	Alive	7	9	7
10	F	*COLQ*	0	3	Y	+	N	Sal	Alive	3	3	3
11	F	*RAPSN*	3	11	N	−	Y	Py	Alive	28	3	2
12	M	*MUSK*	2	11	N	NP	N	Sal	Alive	29	8	5
13	F	*AGRN*	2	3	Y	NP	N	Sal	Alive	21	12	4
14	M	*AGRN*	0	2	Y	+	N	Sal	Alive	3	12	12
15	M	*LRP4*	0	12	Y	+	N	Sal	Alive	17	12	11
16	F	*DOK7*	36	51	N	+	N	Sal	Alive	12	4	3
17	M	*DOK7*	0	77	N	−	Y	N	Alive	36	NA	NA
18	M	*DOK7*	0	23	N	+	N	Sal	Alive	5	8	4
19	M	*CHRNB1*	0	77	N	NP	Y	Py	Alive	28	6	4
20	M	*CHRNB1*	15	40	N	−	N	Py	Alive	54	5	3
21	M	*CHRND*	0	95	N	+	N	Py + Sal	Alive	41	8	6
22	F	*CHRNG*	0	38	N	NP	N	Py	Alive	96	7	3
23	M	*CHRNE*	7	25	N	+	N	Py	Alive	53	3	2
24	M	*CHRNE*	2	27	N	+	N	Py	LTFU	NA	NA	NA
25	F	*CHRNE*	2	35	N	−	N	Py	Alive	79	3	2
26	F	*CHRNE*	24	93	N	+	N	Py	Alive	62	4	2
27	F	*CHRNE*	0	13	N	+	Y	Sal	Alive	1	7	6
28	M	*CHRNA1*	1	4	Y	NP	Y	N	Deceased	NA	NA	NA
29	F	*CHRNA1*	4	15	N	NP	N	Py	Alive	27	4	3
30	M	*SCN4A*	0	1	Y	NP	N	N	Alive	6	NA	NA
31	F	*GMPPB*	111	120	N	NP	N	N	Alive	11	NA	NA
32	F	*GMPPB*	18	51	N	+	N	Py	Alive	55	3	1
33	F	*DPAGT1*	3	65	N	+	N	Py	Alive	79	5	4
34	F	*GFPT1*	12	68	N	NP	N	Py	Alive	42	3	2
35	M	*GFPT1*	60	166	N	+	Y	Py + Sal	Alive	37	7	4
36	F	*GFPT1*	108	126	N	+	Y	Py	Alive	3	3	2

*Note:* Neonatal onset was coded as 0 months. + = positive; − = negative.

Abbreviations: F, female; LTFU, lost to follow‐up; M, male; N, no; NA, not available; NP, not performed; Py, pyridostigmine; RNS, repetitive nerve stimulation; Sal, salbutamol; Y, yes.

^a^ MG‐ADL, Myasthenia Gravis‐Activities of Daily Living Scale score at diagnosis.

^b^ MG‐ADL, Final Myasthenia Gravis‐Activities of Daily Living Scale score.

### 2.6 Statistical Analysis

Continuous variables are presented as median and interquartile range (IQR), and categorical variables as counts and percentages (*n* [%]). Age at onset was recorded in months, with neonatal onset coded as 0 months. Baseline and last follow‐up MG‐ADL scores were compared using the Wilcoxon signed‐rank test. Change in MG‐ADL (ΔMG‐ADL) was defined as the difference between follow‐up and baseline scores, with negative values indicating improvement. Fisher's exact test was used to compare categorical outcomes, including ventilator use and early mortality, between treatment or genotype groups. Two‐sided *p* values < 0.05 were considered statistically significant. All analyses were performed using SPSS.

## 3 Results

### 3.1 Clinical Manifestations

**FIGURE 2:**
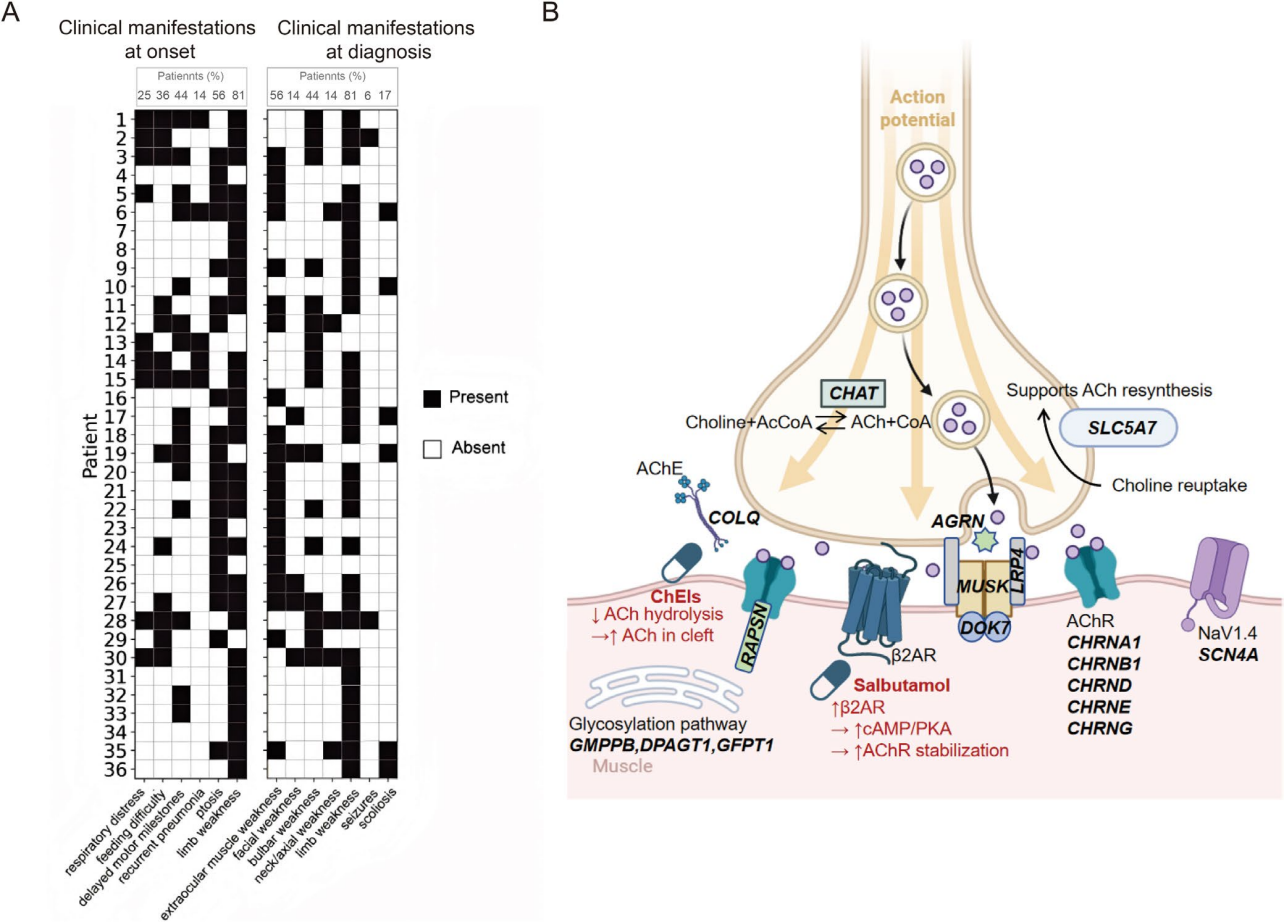
(A) Binary matrix plot of clinical manifestations in 36 patients at symptom onset (left) and at diagnosis (right). Rows represent individual patients, and columns represent clinical features. Black squares indicate the presence of a feature, whereas white squares indicate its absence. At symptom onset, overlapping manifestations included respiratory distress, feeding difficulty, delayed motor milestones, recurrent pneumonia, ptosis, and limb weakness. At diagnosis, examination demonstrated multi‐regional involvement, including extraocular, facial, bulbar, neck/axial, and limb weakness; seizures and scoliosis were observed in a subset of patients. (B) Localization of cohort genes at the neuromuscular junction (NMJ) and sites of action of common therapies. Presynaptic genes include *CHAT* and *SLC5A7*; the synaptic gene is *COLQ*; postsynaptic signaling and receptor‐clustering components include the AGRN–LRP4–MUSK–DOK7 pathway and RAPSN, as well as AChR subunit genes (*CHRNA1*, *CHRNB1*, *CHRND*, *CHRNE*, and *CHRNG*). *SCN4A* encodes the skeletal muscle voltage‐gated sodium channel NaV1.4. Glycosylation‐pathway genes include *GMPPB*, *DPAGT1*, and *GFPT1*. AChE catalytic subunits are attached to the collagen‐like tail subunit *COLQ*, which anchors AChE within the synaptic basal lamina. Cholinesterase inhibitors (ChEIs) increase acetylcholine (ACh) in the synaptic cleft by inhibiting AChE. Salbutamol, a β_2_‐adrenergic agonist, activates β_2_‐adrenergic receptors and downstream cAMP/PKA signaling, which may promote AChR clustering and stabilization at the postsynaptic membrane. The image was created using https://www.biorender.com/.

The cohort comprised 36 patients, including 20 males and 16 females. Age at onset was concentrated in early life: 16 patients (44%, 16/36) presented at birth, 12 (33%, 12/36) between 0 and 1 year, 5 (14%, 5/36) between 1 and 3 years, 1 (3%, 1/36) between 3 and 6 years, and 2 (6%, 2/36) after 6 years of age. By contrast, the median age at diagnosis was 24 months (IQR, 11–66 months; range, 1–166 months) (Table 1). Figure 2A summarizes the distribution of clinical manifestations at symptom onset and at diagnosis, while the corresponding patient‐level presenting complaints and clinical findings are provided in Table S3. At symptom onset, clinical manifestations frequently overlapped: respiratory distress in 9 patients (25%, 9/36), feeding difficulty in 13 (36%, 13/36), delayed motor milestones in 16 (44%, 16/36), recurrent pneumonia in 5 (14%, 5/36), ptosis in 20 (56%, 20/36), and limb weakness in 29 (81%, 29/36). Overall, 28 patients (78%, 28/36) showed multiple clinical manifestations, only 8 patients (22%, 8/36) presented with a single symptom at onset. At diagnosis, extraocular muscle weakness was present in 20 patients (56%, 20/36), facial weakness in 5 (14%, 5/36), bulbar involvement in 16 (44%, 16/36), neck/axial weakness in 5 (14%, 5/36), and limb weakness (proximal and/or distal) in 29 (81%, 29/36). Scoliosis was documented in 6 (17%, 6/36), and seizures occurred in 2 (6%, 2/36).

Patients P7 (*COLQ*), P8 (*COLQ*), P31 (*GMPPB*), P32 (*GMPPB*), P33 (*DPAGT1*), P34 (*GFPT1*), and P36 (*GFPT1*) presented with isolated limb‐girdle weakness at diagnosis. Limb‐girdle weakness has been reported in association with several CMS‐related genes, including *DOK7*, *RAPSN*, *GFPT1*, *GMPPB*, and *DPAGT1* [11, 12]. Notably, isolated limb‐girdle weakness was also observed in two patients with *COLQ* variants in our cohort. P6 and P8, despite coming from two completely unrelated families, both harbor a homozygous variant of *COLQ* (c.679C>T, p.Arg227Ter). However, P6 exhibited multiregional muscle weakness, whereas P8 presented only with limb‐girdle weakness. This underscores the clinical variability and incomplete genotype–phenotype correlation commonly observed in genetic diseases, where the same mutation may result in diverse clinical manifestations influenced by additional genetic, environmental, or epigenetic factors.

### 3.2 Family History

Among the 36 patients, 8 (P6, P11, P17, P19, P27, P28, P35, P36) reported a positive family history. The fathers of P11 and P27 each had a history of childhood ptosis, and genetic testing confirmed that both patients inherited pathogenic variants from their respective fathers. Four patients (P6, P17, P19, P28) had siblings with symptoms suggestive of CMS; however, these individuals were not diagnosed prior to death, and prenatal diagnosis was not performed in subsequent pregnancies. A pair of siblings (P35 and P36) with CMS due to homozygous *GFPT1* variants (c.331C>T, p.Arg111Cys) are currently 17 and 11 years old. Both exhibit limb weakness and scoliosis, but P35 had an earlier onset and additionally exhibited involvement of the neck muscles and extraocular muscles, with more severe symptoms compared to P36. Consequently, P35 was treated with a combination of salbutamol and pyridostigmine, whereas P36, who had milder symptoms, was treated with pyridostigmine alone. This underscores the importance of tailoring treatment to the individual, even in patients with the same genetic variant.

**FIGURE 3:**
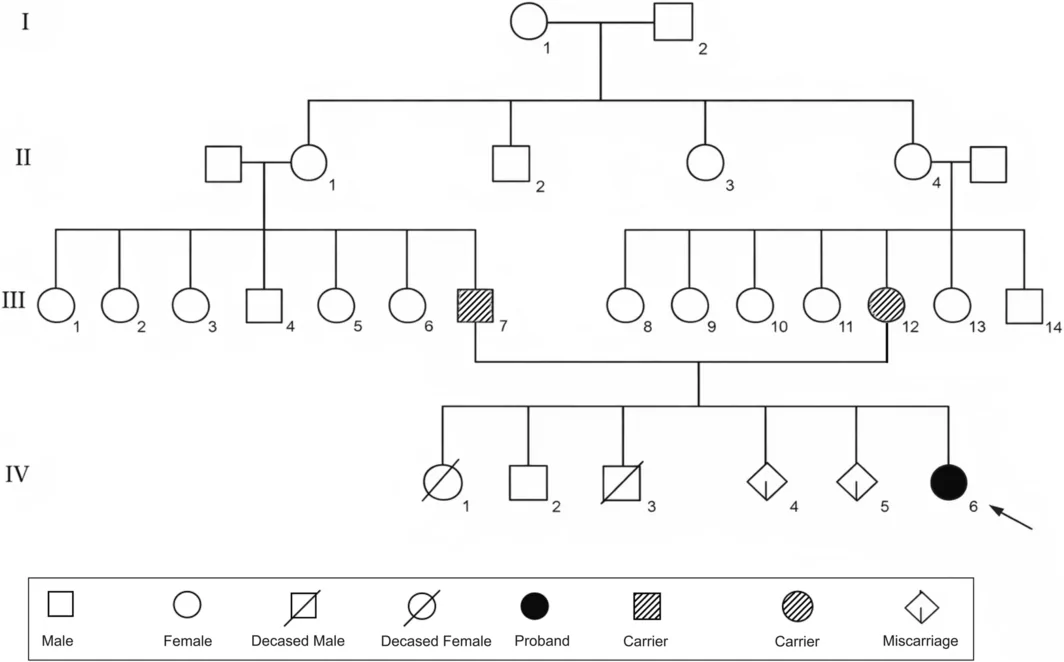
Pedigree of Patient 6. This is a consanguineous family. The proband's paternal grandmother (II‐1) and maternal grandmother (II‐4) are biological sisters. The proband's father (III‐7) and mother (III‐12) have a total of six children. The first child (IV‐1), a girl, exhibited delayed motor development and limb weakness from infancy, and died suddenly at age 8 following an infection. The second child (IV‐2) was a healthy boy. The third child (IV‐3), a boy, presented with limb weakness at birth and died at 4 months from respiratory distress due to pneumonia. The fourth (IV‐4) and fifth (IV‐5) pregnancies resulted in unexplained miscarriages. The sixth child (IV‐6), the proband, was found to have a homozygous *COLQ* variant (c.679C>T, p.Arg227Ter), with the variant inherited from both the father and the mother. Individuals IV‐1 and IV‐3, and pregnancies IV‐4 and IV‐5, were considered possibly affected based on the clinical history, genetic testing and autopsy were not performed. No other family members exhibited weakness, nor did they undergo genetic testing. Family pedigree was generated using Visual Paradigm Online (https://online.visual‐paradigm.com/).

As shown in Figure 3, P6 (IV‐6), a child of the first‐cousin union, carries a homozygous *COLQ* variant (c.679C>T, p.Arg227Ter). Two siblings had CMS‐like symptoms and died after infection‐related deterioration: IV‐1 died suddenly at age 8 following infection, and IV‐3 died at 4 months from pneumonia‐associated respiratory distress. Two additional pregnancies, IV‐4 and IV‐5, resulted in unexplained miscarriages. The patient was started on salbutamol, gradually titrated to 0.1 mg/kg/day without apparent adverse effects. At the most recent follow‐up (age 6 years), P6 attends school and participates in play activities without restriction, but still requires assistance with heavy lifting and climbing stairs.

### 3.3 Gene Spectrum Across NMJ Categories

In this cohort, variants were identified in 17 CMS‐associated genes, encompassing defects at the presynaptic membrane, synaptic cleft, postsynaptic membrane, and those involved in congenital defects of glycosylation. The specific roles of these genes within the context of NMJ are shown in Figure 2B. Postsynaptic CMS accounted for the majority of cases (55.6%, 20/36). The most frequent genes were *COLQ* (13.9%, 5/36), *CHRNE* (13.9%, 5/36), and *CHAT* (11.1%, 4/36), reflecting a relatively balanced distribution rather than predominance of a single gene. As shown in Table S4, biallelic rare variants were identified in 17 CMS‐associated genes spanning presynaptic (*CHAT*, *SLC5A7*), synaptic (*COLQ*), postsynaptic (*CHRNE*, *CHRNA1*, *CHRNB1*, *DOK7*, *AGRN*, *LRP4*, *MUSK*, *RAPSN*, *CHRND*, *CHRNG*), and glycosylation‐related pathways (*GFPT1*, *GMPPB*, *DPAGT1*). Seventeen patients had biallelic pathogenic/likely pathogenic variants (definitive genetic CMS), 11 had one pathogenic/likely pathogenic variant plus one VUS, and 8 (P4, P5, P13, P14, P15, P21, P28, P29) had biallelic VUS. The VUS identified in our cohort exhibited generally low allele frequencies across different populations. The average total population frequency (Total_AF) was 2.1 × 10^−5^, indicating that these variants are rare in the general population. Most of the variants showed frequencies close to zero in the East Asian population (EAS_AF), and the global 95% population frequency (FAF95) was also relatively low, with an average of 3.5 × 10^−5^. This suggests that the VUS are likely rare, though their pathogenic effects remain uncertain.

We next summarize key clinical features observed in our cohort, organized by NMJ localization and the corresponding disease genes.

#### 3.3.1 Presynaptic CMS

*CHAT* is the third most frequent gene identified in our cohort of 36 patients, with 4 patients carrying *CHAT* gene mutations. *CHAT* encodes choline acetyltransferase, and impaired acetylcholine synthesis can lead to early‐life neuromuscular transmission failure. Prior studies report that a substantial proportion of affected infants (approximately 84%) present at birth with respiratory pauses or respiratory distress, which may be misinterpreted clinically as epileptic seizures [13]. Because pyridostigmine can reduce the frequency of respiratory pauses, improve respiratory symptoms, and decrease ventilatory requirements thereby potentially limiting secondary hypoxic brain injury, it is currently recommended as first‐line therapy for *CHAT*‐CMS [14].

In our cohort, 3 out of 4 patients with *CHAT* variants (P1–P3) developed severe infection‐associated respiratory deterioration. Despite pyridostigmine being initiated immediately after diagnosis, these patients passed away because of respiratory failure or the family discontinuing treatment. P4 presented with early‐onset weakness and ptosis but did not experience respiratory pauses or distress and had normal cognitive development. Pyridostigmine was initiated at 4 years of age, and by age 7, the patient was independent in daily activities, although exertional fatigability persisted. This case highlights that when respiratory failure is not present, the initiation of pyridostigmine can significantly improve clinical outcomes.

Further analysis revealed that *CHAT*‐CMS patients experienced significantly worse outcomes compared to non‐*CHAT* patients, with a notably higher rate of ventilator use. Specifically, 3 out of 4 patients with *CHAT* mutations required ventilator support, compared to only 6 out of 32 non‐*CHAT* patients (*p* = 0.041). This potentially underscores that the *CHAT* gene variant is associated with higher ventilator dependency.

#### 3.3.2 Synaptic CMS

*COLQ* is one of the most frequent genes identified in our cohort. *COLQ* encodes the collagen‐like tail subunit of acetylcholinesterase (AChE), which anchors AChE at the neuromuscular junction; disruption of this process impairs acetylcholine clearance and neuromuscular transmission, leading to weakness [4]. Prior studies suggest that infantile‐onset *COLQ*‐CMS tends to be more severe than childhood‐onset disease. Because ChEIs may be ineffective or detrimental in this subtype, β_2_‐adrenergic agonists such as salbutamol are commonly used as a mechanism‐aligned therapeutic option [1].

In our cohort, synaptic cleft involvement was observed exclusively in patients with *COLQ* variants (13.9%, 5/36). All 5 presented at birth with generalized weakness; two of the five *COLQ* patients developed scoliosis, which is consistent with findings in the literature. Kaya et al. [15] reported that scoliosis is more frequently associated with CMS patients carrying *COLQ* variants, emphasizing its potential as an early clinical indicator. While scoliosis in CMS patients is not universally severe, it is important to recognize it as a symptom that may warrant early intervention. In our 2 *COLQ* patients with scoliosis, salbutamol and rehabilitation therapy were administered, which resulted in favorable outcomes. Currently, they do not require surgical intervention, and their daily activities are not significantly affected by the scoliosis. We will continue to monitor their long‐term prognosis closely. These findings underscore the importance of early genetic diagnosis and intervention to address potential complications like scoliosis, ensuring better management and long‐term outcomes for CMS patients.

#### 3.3.3 Postsynaptic CMS

Postsynaptic CMS was the most common category in our cohort (55.6%, 20/36). *CHRNE*, a leading CMS gene worldwide, encodes the ε subunit of the adult acetylcholine receptor (AChR), and variants can impair receptor function and neuromuscular transmission [1, 16, 17, 18, 19]. AChR defects are classically divided into slow‐channel syndrome (SCS) and fast‐channel syndrome (FCS), which differ in both clinical course and treatment. SCS is associated with prolonged channel opening and is typically managed with open‐channel blockers, while FCS reflects reduced channel opening or function and is commonly treated with ChEIs [17, 20]. These distinctions make accurate subtype recognition clinically important. In our cohort, 5 patients (P23–P27) carried *CHRNE* variants. Four patients (P23–P26) were classified as FCS and treated with pyridostigmine, whereas one (P27) was classified as SCS and received salbutamol. All FCS patients showed improvement on longitudinal MG‐ADL assessment, while the SCS patient remained stable after 3 months of treatment.

The agrin‐LRP4‐MuSK‐DOK7 signaling axis is essential for neuromuscular junction development by coordinating AChR clustering and postsynaptic differentiation [21, 22]. *LRP4*‐CMS has been reported to present with variable severity, ranging from perinatal respiratory and feeding compromise to later fatigable limb‐girdle weakness, and many patients show a decrement on RNS [23, 24, 25, 26]. In our cohort, P15 carried *LRP4* variants c.186T>G (p.Asp62Glu) and c.3697G>A (p.Glu1233Lys), with both classified as VUS. However, the overall pattern—recessive inheritance, a CMS‐compatible phenotype with prominent early respiratory involvement, and supportive electrophysiology—provided convergent clinical evidence of NMJ dysfunction. Given this concordance and the potential for mechanism‐aligned benefit, a pragmatic trial of salbutamol was initiated, but was discontinued after 1 month because of limited family adherence. We will continue longitudinal follow‐up and periodic re‐evaluation of variant interpretation as additional clinical and functional evidence accrues. *DOK7* is a major gene within the agrin‐LRP4‐MuSK‐DOk7 pathway, and adult data suggest substantial long‐term morbidity: in a French multicenter cohort, 38.6% experienced ICU‐requiring exacerbations and 36.3% required ventilatory support and were wheelchair‐dependent at last follow‐up. Among our 3 *DOK7* cases (P16–P18), P16 and P17 each harbored compound heterozygous variants, with one variant being a VUS: P16 with c.94G>C (p.Val32Leu) and c.957del (p.Lys320SerfsTer136); P17 with c.‐1_1del (p.Met1?) and c.54+25_55‐38del (p.?). P16 had prominent extraocular muscle weakness and a positive RNS, which supported NMJ involvement and prompted early initiation of salbutamol. In contrast, although P17 also had early‐onset limb weakness and a positive family history, RNS was negative and salbutamol has not yet been started; a muscle biopsy is planned to provide additional pathological/functional evidence to guide treatment decisions and to better characterize long‐term trajectories and treatment responsiveness in this young cohort.

#### 3.3.4 Glycosylation‐Related CMS

Variants in glycosylation‐pathway genes can disrupt neuromuscular junction function, likely by altering the glycosylation, trafficking, and stability of key synaptic proteins (including AChR), and they often manifest with a limb‐girdle pattern of weakness. Tubular aggregates may be observed on muscle pathology, although this feature is not consistently present across patients [27]. In a previously reported Chinese CMS cohort, *GFPT1* variants accounted for 27.8% of cases, suggesting potential population‐specific differences in genetic architecture [28].

In our cohort, glycosylation‐related CMS comprised 6/36 (16.7%) patients, including *GFPT1* (*n* = 3), *GMPPB* (*n* = 2), and *DPAGT1* (*n* = 1). Among patients P31‐P36, 5 of 6 (83.3%, 5/6) presented with isolated limb‐girdle weakness, whereas P35 additionally exhibited extraocular muscle involvement (16.7%, 1/6). Moreover, P35 and P36 were siblings and both had scoliosis, highlighting potential intrafamilial clustering of orthopedic manifestations in this subgroup.

### 3.4 Auxiliary Examinations

RNS provides objective electrophysiological evidence of impaired neuromuscular transmission and is therefore widely used as an adjunctive test in CMS evaluation. RNS was performed in 21 of 36 patients, as shown in Table 1. Sixteen of 21 patients (76.2%, 16/21) demonstrated a ≥ 10% decrement in compound muscle action potential amplitude during low‐frequency stimulation (1, 3, and 5 Hz). These patients harbored variants in *SLC5A7*, *COLQ*, *AGRN*, *LRP4*, *DOK7*, *CHRND*, *CHRNE*, *GMPPB*, *GFPT1*, and *DPAGT1*. The remaining 5 patients (23.8%, 5/21)—P2 (*CHAT*), P11 (*RAPSN*), P17 (*DOK7*), P20 (*CHRNB1*), and P25 (*CHRNE*)—showed no decrement on RNS despite compatible clinical phenotypes and supportive genetic findings. Notably, among the 8 patients with biallelic VUS, RNS was performed in 4 patients (P5, P14, P15, and P21), all of whom demonstrated a decrement; RNS was not performed in the remaining 4 patients. Among the 4 patients who did not undergo RNS, 3 (P4, P13, and P29) had clear clinical myasthenic features; following shared decision‐making with the families, a pragmatic therapeutic trial was undertaken, and all 3 showed a reduction in MG‐ADL scores after treatment initiation. While treatment response alone is not diagnostic, this observation provides supportive evidence of NMJ involvement and adds weight to the overall clinicogenetic interpretation of these biallelic VUS findings.

Among the additional investigations, serum creatine kinase (CK) levels were within the reference range (25–200 U/L) in all patients except P31 (*GMPPB*), in whom serial CK measurements ranged from 1037 to 21,000 U/L. Muscle biopsy revealed only scattered necrotic and regenerating fibers, consistent with nonspecific myopathic changes. The basis for this isolated CK elevation could not be determined within the scope of the present study.

### 3.5 Ventilator Usage

In CMS, the most life‐threatening events usually stem from acute respiratory complications rather than slow disease progression. Ventilator support is therefore most commonly initiated in the neonatal/infant period for respiratory distress, apnea, or ventilatory failure, often triggered by physiological stressors such as infection. The timing and difficulty of ventilator liberation may differ by genotype, prompting the following summary of ventilator usage in our cohort.

A total of 9 patients required ventilator support, including 3 with *CHAT* variants (P1–P3), 1 with *COLQ* variant (P10), 2 with *AGRN* variants (P13–P14), 1 with an *LRP4* variant (P15), 1 with a *CHRNA1* variant (P28), and 1 with an *SCN4A* variant (P30). These patients were placed on ventilators due to respiratory distress shortly after birth or during infancy. Three *CHAT* patients (P1–P3) and the *CHRNA1* patient (P28) died early, whereas the remaining ventilated patients (P10, P13–P15, and P30) were successfully weaned off mechanical ventilation and subsequently breathed independently; overall, 7 of the 9 ventilated patients experienced difficult extubation/weaning requiring repeated intubations. Ventilator use was significantly more frequent in *CHAT*‐CMS than in non‐*CHAT* cases (75.0% [3/4] vs. 18.8% [6/32]; Fisher's exact test, *p* = 0.041), underscoring *CHAT*‐related disease as a high‐risk genotype for early‐life respiratory failure in our cohort.

### 3.6 Treatment and Outcomes

Among the 36 patients, 28 (78%, 28/36) met criteria for a confirmed diagnosis, 6 (17%, 6/36) were classified as probable, and 2 (5%, 2/36) as suspicious. Seventeen patients received pyridostigmine, 10 received salbutamol, and 2 received combined pyridostigmine plus salbutamol; 7 remained untreated due to loss to follow‐up, very young age, or concerns regarding tolerability. Follow‐up from diagnosis to last contact ranged from 1 to 96 months. At the final follow‐up, 4 patients (11%, 4/36) had died and 3 (8%, 3/36) were lost to follow‐up.

Among the remaining 29 patients (81%, 29/36), 2 children continued to have dysphagia requiring nasogastric feeding, while the others were able to perform basic activities of daily living. In patients with paired MG‐ADL assessments, scores improved overall from baseline to last follow‐up (median 5 [IQR 3.25–7.75] vs. 3 [IQR 2–4]; Wilcoxon signed‐rank test, *p* < 0.001).

**FIGURE 4:**
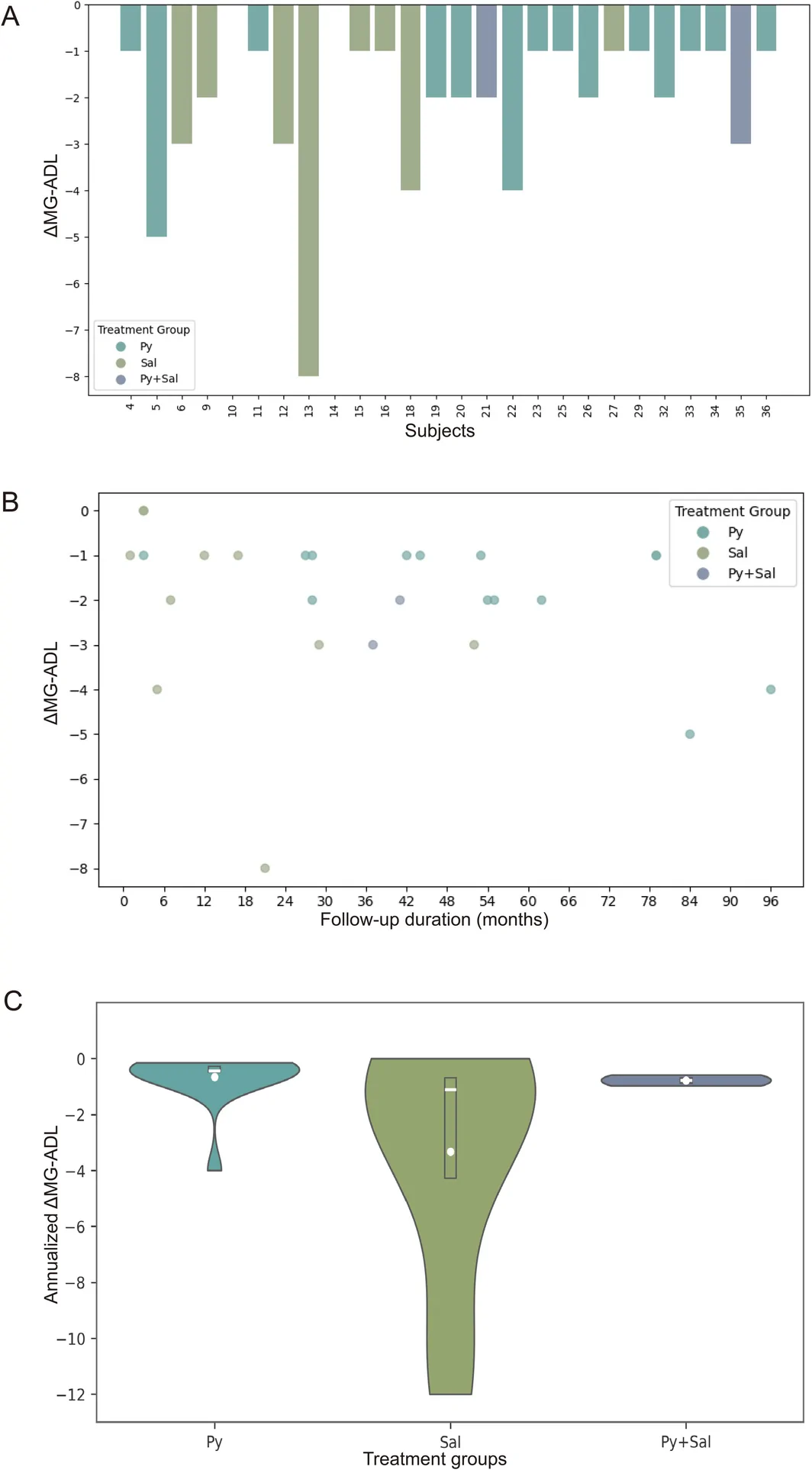
Longitudinal change in MG‐ADL according to treatment group. (A) Waterfall plot showing individual changes in MG‐ADL from baseline to last follow‐up (ΔMG‐ADL = MG‐ADL2 − MG‐ADL1) among patients with paired assessments, ranked by magnitude of change. Negative values indicate improvement. Colors denote treatment group (Py, Sal, or Py + Sal). (B) Scatter plot of follow‐up duration versus ΔMG‐ADL. Negative values indicate improvement. Colors denote treatment group. (C) Violin plots showing annualized change in MG‐ADL change by treatment group, calculated as [(MG‐ADL2 − MG‐ADL1)/follow‐up months × 12] by treatment. Negative values indicate improvement.

Consistent with this improvement, the waterfall plot demonstrated predominantly negative ΔMG‐ADL values across treatment groups, indicating functional improvement in most individuals (Figure 4A). The scatter plot of follow‐up duration versus ΔMG‐ADL showed heterogeneous changes without a clear trend of greater improvement with longer follow‐up (Figure 4B). After annualization, changes in MG‐ADL were largely negative across treatment groups, consistent with overall improvement. The salbutamol group showed wider dispersion, partly driven by patients with short follow‐up durations, whereas interpretation of the pyridostigmine‐plus‐salbutamol group was limited by the small sample size (Figure 4C).

## 4 Discussion

In this single‐center cohort of 36 Chinese pediatric patients evaluated for congenital myasthenic syndromes (CMS), we observed substantial clinical and genetic heterogeneity with a clear early‐life clustering of onset. Most children presented at birth or within the first year of life, typically with overlapping and relatively nonspecific features—limb weakness, ocular and/or bulbar involvement, hypotonia with delayed motor development, feeding difficulty, and infection‐associated worsening—so that “classic” fluctuating fatigable weakness was often subtle or atypical in neonates and infants, increasing the risk of delayed recognition [1, 4, 6].

The molecular spectrum spanned 17 CMS‐associated genes across presynaptic, synaptic, postsynaptic, and glycosylation‐related pathways, with postsynaptic defects representing the largest subgroup. As reported in other populations, the relative contribution of individual genes is likely shaped by ancestry, referral patterns, and cohort composition [2, 5, 7, 28]. Our data therefore add pediatric evidence from China and underscore that gene‐frequency expectations derived from large international series may not generalize uniformly across settings. Phenotypic variability was also evident within genotypes, reinforcing the need to interpret genetic findings in conjunction with clinical course and ancillary testing rather than as a stand‐alone predictor of severity.

In our cohort, CMS treatment selection was subtype‐ and mechanism‐informed, supporting a precision‐based therapeutic approach. Pyridostigmine was selected as first‐line therapy for presynaptic CMS (notably *CHAT*) and for several postsynaptic AChR‐deficiency phenotypes, whereas salbutamol was preferentially used for *COLQ*‐related synaptic AChE deficiency, consistent with established subtype‐specific pharmacologic considerations. Notably, our *CHRNE* cases illustrate that gene identity alone is insufficient for prescribing: distinct channel kinetics within the same gene can imply different therapeutic strategies, with pyridostigmine used for the FCS phenotype and salbutamol for the SCS phenotype in our cohort. Overall, our data support a mechanism‐guided strategy in which genetic results are interpreted with clinical and electrophysiologic data to choose and titrate therapy. Treatment decisions should therefore be based on genetic findings interpreted alongside clinical and electrophysiologic evidence. Accurate and timely identification of CMS is equally important for medication safety, because a range of agents—including aminoglycosides, macrolides, fluoroquinolones, magnesium salts, β‐blockers, certain antiarrhythmic drugs, and neuromuscular blocking agents—may further impair neuromuscular transmission. These drugs should be avoided whenever possible or, when clinically necessary, administered with close monitoring; penicillamine and interferons are considered contraindicated in disorders of the neuromuscular junction [29]. Particular caution is therefore warranted when antimicrobial treatment is required during an intercurrent infection.

A key finding with immediate clinical relevance was the disproportionately severe respiratory course in *CHAT*‐CMS. Overall, 9/36 (25.0%) patients required mechanical ventilation, typically initiated shortly after birth or during infancy, and weaning was frequently difficult (7/9 required repeated intubations), highlighting limited respiratory reserve during intercurrent infections in early‐onset CMS. Ventilator use was significantly more common in *CHAT*‐CMS than in non‐*CHAT* cases (75.0% [3/4] vs. 18.8% [6/32]; Fisher's exact test *p* = 0.041). Early mortality also clustered in *CHAT*: 3/4 *CHAT* patients died compared with 1/32 non‐*CHAT* patients (Fisher's exact test *p* = 0.002). Together, these data support *CHAT*‐CMS as a high‐risk genotype in pediatric practice, warranting earlier recognition, anticipatory infection planning, and close respiratory monitoring and counseling.

Variant interpretation was complicated by the high prevalence of VUS, a common challenge in pediatric genomic diagnostics. In this setting, VUS findings should be interpreted in the context of the full clinical and electrophysiologic phenotype, and should be prioritized for periodic reinterpretation and genomic reanalysis as population resources, case evidence, and gene–disease knowledge evolve. Electrophysiology provided supportive—though not definitive—functional evidence: RNS was feasible in 21/36 patients, and 16/21 (76.2%) showed a ≥ 10% decrement. Among the 8 patients with biallelic VUS, RNS data were available for 4 (P5, P14, P15, and P21), all of whom demonstrated a decrement; the remaining four had no RNS data. Of these four, three showed MG‐ADL improvement after a shared decision‐making process and a therapeutic trial. Together, these findings support a pragmatic clinicogenetic approach in which phenotype–electrophysiology concordance informs provisional diagnosis and management, coupled with longitudinal follow‐up and scheduled reanalysis of VUS. Because RNS can be insensitive in young children due to technical and cooperation limitations, repeat RNS should be considered during follow‐up when testing conditions are more optimal.

However, this study has some limitations. First, as a single‐center study with a relatively small sample size, the findings may not fully represent the broader diversity of CMS patients across different regions in China. Second, some patients could not undergo complete electrophysiological testing due to their age, limiting the data's comprehensiveness. Third, the high prevalence of VUS complicates genetic interpretation, and functional validation studies were not performed for these variants, limiting definitive assessment of their pathogenicity. In addition, we currently lack sufficient long‐term follow‐up data to confirm the clinical significance of these variants. Finally, follow‐up was limited in some patients, restricting the evaluation of long‐term clinical outcomes and treatment responses.

## 5 Conclusions

In conclusion, CMS should remain high on the differential diagnosis for neonates and infants presenting with feeding or respiratory difficulties, hypotonia with motor delay, and infection‐triggered deterioration. Given the genetic and phenotypic diversity of CMS, personalized treatment is crucial, particularly for high‐risk genotypes like *CHAT*, which require close respiratory management. An integrated phenotype–electrophysiology–genomics approach is particularly valuable for early diagnosis and personalized treatment.

## Author Contributions

Conceptualization: Hui Xiong; Methodology: Lin Ge and Kaiyue Ma; Investigation: Bingbing Jia, Wenna Ma, and Lei Yang; Data curation: Bingbing Jia and Xiaona Fu; Resources: Zhimei Liu, Wenmiao Xu, Lijuan Wang and Guangyuan Zhao; Formal analysis: Liya Cui and Xiaona Fu; Supervision: Jiuwei Li, Jie Wu, Quan Wang and Hui Xiong; Project Administration: Junlan Lv, Changhong Ding and Xiaotun Ren; Writing – original draft preparation: Liya Cui; Writing‐review and editing: Hui Xiong, Kaiyue Ma and Xinming Shen.

## Funding

This work was supported by the National Natural Science Foundation of China (No. 82471430), and the High Innovation Plan (No. G202512059).

## Disclosure

All authors read and approved the final manuscript.

## Ethics Statement

This study was conducted in accordance with the Declaration of Helsinki (2013 revision) and approved by the Institutional Review Board of Beijing Children's Hospital, Capital Medical University (No. [2025]‐E‐166‐R). The requirement for written informed consent was waived by the Institutional Review Board due to the retrospective design and the use of de‐identified clinical data. Patient confidentiality was strictly maintained, and no identifiable information is included in this manuscript. Sex was recorded for all participants and included in descriptive analyses; however, sex‐stratified analyses were not performed due to sample size limitations.

## Conflicts of Interest

The authors declare no conflicts of interest.

## Supporting information


**Table S1:** Panel gene list.
**Table S2:** Variant‐level annotation of DNA variants identified in the cohort.
**Table S3:** Patient‐level presenting complaints at symptom onset and clinical findings at diagnosis.
**Table S4:** Patient‐level variants and inheritance in 36 patients.

## Data Availability

The data supporting the findings of this study are not publicly available due to patient privacy and ethical restrictions. De‐identified data may be made available from the corresponding author upon reasonable request and subject to institutional approval.

## References

[1] A. G. Engel , X. M. Shen , D. Selcen , and S. M. Sine , “Congenital Myasthenic Syndromes: Pathogenesis, Diagnosis, and Treatment,” Lancet Neurology 14 (2015): 420–434, 10.1016/S1474-4422(14)70201-7. 25792100 PMC4520251

[2] J. Theuriet , M. Masingue , A. Behin , et al., “Congenital Myasthenic Syndromes in Adults: Clinical Features, Diagnosis and Long‐Term Prognosis,” Brain 147 (2024): 3849–3862, 10.1093/brain/awae124. 38696726 PMC11531845

[3] K. Ohno , M. Ito , and B. Ohkawara , “Review of 40 Genes Causing Congenital Myasthenic Syndromes,” Journal of Human Genetics (2025) Online ahead of print, 10.1038/s10038-025-01355-9. 40533459

[4] K. Ohno , B. Ohkawara , X. M. Shen , D. Selcen , and A. G. Engel , “Clinical and Pathologic Features of Congenital Myasthenic Syndromes Caused by 35 Genes: A Comprehensive Review,” International Journal of Molecular Sciences 24 (2023): 3730, 10.3390/ijms24043730. 36835142 PMC9961056

[5] A. Troha Gergeli , D. Neubauer , T. Golli , et al., “Prevalence and Genetic Subtypes of Congenital Myasthenic Syndromes in the Pediatric Population of Slovenia,” European Journal of Paediatric Neurology 26 (2020): 34–38, 10.1016/j.ejpn.2020.02.002. 32070632

[6] J. Finsterer , “Congenital Myasthenic Syndromes,” Orphanet Journal of Rare Diseases 14 (2019): 57, 10.1186/s13023-019-1025-5. 30808424 PMC6390566

[7] S. Ramdas and D. Beeson , “Congenital Myasthenic Syndromes: Where Do We Go From Here?,” Neuromuscular Disorders 31 (2021): 943–954, 10.1016/j.nmd.2021.07.400. 34736634

[8] S. Spendiff , H. Lochmüller , and R. A. Maselli , “Congenital Myasthenic Syndromes,” International Review of Neurobiology 182 (2025): 253–274, 10.1016/bs.irn.2025.04.025. 40675739

[9] M. Takhman , R. Shihab , M. Hattab , et al., “Salbutamol in Congenital Myasthenic Syndrome: A Systematic Review,” BMC Neurology 26 (2026): 82, 10.1186/s12883-025-04569-8. PMC12888253 41382066

[10] S. Nicolau and M. Milone , “The Electrophysiology of Presynaptic Congenital Myasthenic Syndromes With and Without Facilitation: From Electrodiagnostic Findings to Molecular Mechanisms,” Frontiers in Neurology 10 (2019): 257, 10.3389/fneur.2019.00257. 30941097 PMC6433874

[11] C. W. Lam , K. S. Wong , H. W. Leung , et al., “Limb Girdle Myasthenia With Digenic RAPSN and a Novel Disease Gene AK9 Mutations,” European Journal of Human Genetics 25 (2016): 192–199, 10.1038/ejhg.2016.162. 27966543 PMC5255960

[12] T. Evangelista , M. Hanna , and H. Lochmüller , “Congenital Myasthenic Syndromes With Predominant Limb Girdle Weakness,” Journal of Neuromuscular Diseases 2 (2015): S21–S29, 10.3233/JND-150098. PMC4746746 26870666

[13] K. Ohno , A. Tsujino , J. M. Brengman , et al., “Choline Acetyltransferase Mutations Cause Myasthenic Syndrome Associated With Episodic Apnea in Humans,” Proceedings of the National Academy of Sciences of the United States of America 98 (2001): 2017–2022, 10.1073/pnas.98.4.2017. 11172068 PMC29374

[14] P. Arican , P. Gencpinar , D. Cavusoglu , and N. Olgac Dundar , “Clinical and Genetic Features of Congenital Myasthenic Syndromes due to CHAT Mutations: Case Report and Literature Review,” Neuropediatrics 49 (2018): 283–288, 10.1055/s-0038-1654706. 29783273

[15] O. Kaya and S. Kirik , “Can Scoliosis Help the Early Diagnosis of Congenital Myasthenic Syndrome?,” Cureus 15 (2023): e45875, 10.7759/cureus.45875. 37766777 PMC10520996

[16] J. R. Parr , M. J. Andrew , M. Finnis , D. Beeson , A. Vincent , and S. Jayawant , “How Common Is Childhood Myasthenia? The UK Incidence and Prevalence of Autoimmune and Congenital Myasthenia,” Archives of Disease in Childhood 99 (2014): 539–542, 10.1136/archdischild-2013-304788. 24500997

[17] K. Yang , H. Cheng , F. Yuan , et al., “CHRNE Compound Heterozygous Mutations in Congenital Myasthenic Syndrome,” Medicine (Baltimore) 97 (2018): 0347, 10.1097/MD.0000000000010347. PMC5944527 29702980

[18] A. Della Marina , A. Koutsoulidou , D. Natera‐de Benito , et al., “Blood Biomarker Fingerprints in a Cohort of Patients With CHRNE‐Related Congenital Myasthenic Syndrome,” Acta Neuropathologica Communications 13 (2025): 29, 10.1186/s40478-025-01946-9. 39948634 PMC11823195

[19] K. Huang , Y. B. Luo , F. F. Bi , and H. Yang , “Pharmacological Strategy for Congenital Myasthenic Syndrome With CHRNE Mutations: A Meta‐Analysis of Case Reports,” Current Neuropharmacology 19 (2021): 718–729, 10.2174/1570159X18666200729092332. 32727330 PMC8573743

[20] A. Chaouch , J. S. Müller , V. Guergueltcheva , et al., “A Retrospective Clinical Study of the Treatment of Slow‐Channel Congenital Myasthenic Syndrome,” Journal of Neurology 259 (2011): 474–481, 10.1007/s00415-011-6204-9. 21822932

[21] Y. Zong , B. Zhang , S. Gu , et al., “Structural Basis of Agrin‐LRP4‐MuSK Signaling,” Genes & Development 26 (2012): 247–258, 10.1101/gad.180885.111. 22302937 PMC3278892

[22] T. Xie , G. Xu , Y. Liu , et al., “Structural Insights Into the Assembly of the Agrin/LRP4/MuSK Signaling Complex,” Proceedings of the National Academy of Sciences of the United States of America 120 (2023): e2300453120, 10.1073/pnas.2300453120. 37252960 PMC10266037

[23] B. Ohkawara , M. Cabrera‐Serrano , T. Nakata , et al., “LRP4 Third β‐Propeller Domain Mutations Cause Novel Congenital Myasthenia by Compromising Agrin‐Mediated MuSK Signaling in a Position‐Specific Manner,” Human Molecular Genetics 23 (2013): 1856–1868, 10.1093/hmg/ddt578. 24234652 PMC3943522

[24] A. Barik , Y. Lu , A. Sathyamurthy , et al., “LRP4 is Critical for Neuromuscular Junction Maintenance,” Journal of Neuroscience 34 (2014): 13892–13905, 10.1523/JNEUROSCI.1733-14.2014. 25319686 PMC4198535

[25] T. Al Jabry , N. Al‐Hashmi , B. Abdelhadi , et al., “LRP4 Site‐Specific Variants in the Third β‐Propeller Domain Cause Congenital Myasthenic Syndrome Type 17,” European Journal of Medical Genetics 67 (2024): 104903, 10.1016/j.ejmg.2023.104903. 38101565

[26] B. H. Chen , Z. Y. Lin , X. X. Zeng , Y. H. Jiang , and F. Geng , “LRP4‐Related Signalling Pathways and Their Regulatory Role in Neurological Diseases,” Brain Research 1825 (2024): 148705, 10.1016/j.brainres.2023.148705. 38065285

[27] J. Senderek , J. S. Müller , M. Dusl , et al., “Hexosamine Biosynthetic Pathway Mutations Cause Neuromuscular Transmission Defect,” American Journal of Human Genetics 88 (2011): 162–172, 10.1016/j.ajhg.2011.01.008. 21310273 PMC3035713

[28] Y. Zhao , Y. Li , Y. Bian , et al., “Congenital Myasthenic Syndrome in China: Genetic and Myopathological Characterization,” Annals of Clinical Translational Neurology 8 (2021): 898–907, 10.1002/acn3.51346. 33756069 PMC8045908

[29] M. A. M. Salih and P. B. Kang , “Neuromuscular Transmission Disorders,” in Clinical Child Neurology, ed. M. A. M. Salih (Springer Nature, 2020), 1257–1279, 10.1007/978-3-319-43153-6_42.

